# Prediction of Heart Disease Based on Machine Learning Using Jellyfish Optimization Algorithm

**DOI:** 10.3390/diagnostics13142392

**Published:** 2023-07-17

**Authors:** Ahmad Ayid Ahmad, Huseyin Polat

**Affiliations:** 1 Computer Engineering Department, Gazi University, Ankara 06560, Turkey; polath@gazi.edu.tr; 2 Information Technology Department, Kirkuk University, Kirkuk 36013, Iraq

**Keywords:** heart disease diagnosis, feature selection, jellyfish optimization, machine learning, SVM

## Abstract

Heart disease is one of the most known and deadly diseases in the world, and many people lose their lives from this disease every year. Early detection of this disease is vital to save people’s lives. Machine Learning (ML), an artificial intelligence technology, is one of the most convenient, fastest, and low-cost ways to detect disease. In this study, we aim to obtain an ML model that can predict heart disease with the highest possible performance using the Cleveland heart disease dataset. The features in the dataset used to train the model and the selection of the ML algorithm have a significant impact on the performance of the model. To avoid overfitting (due to the curse of dimensionality) due to the large number of features in the Cleveland dataset, the dataset was reduced to a lower dimensional subspace using the Jellyfish optimization algorithm. The Jellyfish algorithm has a high convergence speed and is flexible to find the best features. The models obtained by training the feature-selected dataset with different ML algorithms were tested, and their performances were compared. The highest performance was obtained for the SVM classifier model trained on the dataset with the Jellyfish algorithm, with Sensitivity, Specificity, Accuracy, and Area Under Curve of 98.56%, 98.37%, 98.47%, and 94.48%, respectively. The results show that the combination of the Jellyfish optimization algorithm and SVM classifier has the highest performance for use in heart disease prediction.

## 1. Introduction

According to the World Health Organization, despite significant advances in diagnosis and treatment, mortality from heart disease remains the leading cause of death worldwide, accounting for about one-third of annual deaths [1]. “Heart disease” is a general term used to describe a group of heart conditions and diseases, including Coronary Artery Disease, Arrhythmia, Heart Valve Disease, and Heart Failure, which cause the heart not to pump blood healthily.

The most common type of heart disease is Coronary Artery Disease. The disease is a medical condition in which the coronary arteries that supply blood to the heart muscle become narrowed or blocked due to plaque build-up on their inner walls. This can lead to serious complications such as a heart attack, heart failure, and arrhythmias, as it reduces blood flow to the heart muscle. In some cases, procedures such as angioplasty or bypass surgery may be necessary to improve blood flow to the heart.

The second common heart disease is Arrhythmia. Arrhythmia is caused by disturbances in the normal electrical activity of the heart. The normal beating rhythm of the heart is disrupted because the electrical impulses in the heart responsible for synchronizing the heartbeat are not working properly. As a result, the heartbeat may be faster, slower, or more irregular than normal [2,3]. Millions of people worldwide are affected by Arrhythmia. Symptoms can include a fast or irregular heartbeat, shortness of breath, dizziness or fainting, chest pain or discomfort, fatigue, and weakness. There are many different types of arrhythmias, and some types of arrhythmias are harmless, while others can be life-threatening. While many people may experience occasional episodes of mild arrhythmia in their lives, some people may struggle with more serious types of arrhythmias. For example, a type of Arrhythmia known as Atrial Fibrillation can occur in about 10% of adults over the age of 60 and can increase the risk of stroke. On the other hand, a serious type of Arrhythmia known as Ventricular Fibrillation is considered a cause of heart attacks and can be fatal. Some types of arrhythmias can be inherited, while others can be caused by lifestyle factors or other heart diseases. In most early-diagnosed cases, arrhythmias can be treated. Patients with these disorders are much less likely to die suddenly if they receive prompt, thorough diagnosis and medical care [4,5].

The main reasons for the significant increase in heart disease in recent years are people’s lifestyle, lack of exercise, and consumption of various processed foods. Heart disease in its advanced stages can cause heart attacks and endanger the lives of patients, so it is necessary to detect the disease quickly and in its early stages with intelligent and therapeutic methods. One of the major challenges in the diagnosis of heart disease is the reluctance of patients to participate in clinical trials. On the other hand, the cost of these trials is high, and they take a lot of time, which is why they receive little attention. In contrast to clinical methods for diagnosing heart disease, some methods can be used to analyze the pattern of the disease by analyzing information from patients and healthy people [6].

In recent years, applications of artificial intelligence technology, especially Machine Learning (ML), in the field of auxiliary diagnosis have developed rapidly, and efficient progress has been made in automatic detection applications [7,8,9,10]. The advantage of ML methods is that they can diagnose diseases, such as heart disease, with low-cost and reasonable accuracy [11]. ML techniques for diagnosing heart disease do not require multiple clinical trials, most of which are invasive, and a set of information and features can help to diagnose the disease with high accuracy. It should be noted that although ML technology has made advances in the automatic diagnosis of heart disease, the approval of doctors is still a necessary link in diagnosis and treatment. It is also clear that ML-based disease diagnosis offers an opportunity to increase doctors’ work efficiency and generate economic benefits. In the age of big data, with ever-expanding datasets and the development of new ML algorithms, it is expected that ML applications will undoubtedly have a major impact on automated heart disease prediction [12,13,14,15,16]. In the literature, there are research papers that try to predict heart disease with different datasets and different types of ML algorithms.

Dubey A. K. et al. examined the performance of ML models such as Logistic Regression (LR), Decision Tree (DT), Random Forest (RF), Support Vector Machine (SVM), SVM with grid search (SVMG), K-Nearest Neighbor (KNN) and Naïve Bayes (NB) for heart disease classification. Cleveland and Statlog datasets from the UCI Machine Learning repository were used for training and testing. The experimental results show that LR and SVM classifier models perform better on the Cleveland dataset with 89% accuracy, while LR performs better on the Statlog dataset with 93% accuracy [17].

Karthick K. et al. used SVM, Gaussian Naive Bayes (GNB), LR, LightGBM, XGBoost, and RF algorithms to build an ML model for heart disease risk prediction. In this study, the authors applied the Chi-square statistical test to select the best features from the Cleveland heart disease dataset. After feature selection, the RF classifier model obtained the highest classification accuracy rate of 88.5% [18].

Veisi H. et al. developed various ML models such as DT, RF, SVM, XGBoost, and Multilayer Perceptron (MLP) using the Cleveland heart disease dataset to predict heart disease. Various preprocessing (outlier detection, normalization, etc.) and feature selection processes were applied to the dataset. Among the ML models evaluated, the highest accuracy of 94.6% was achieved using the MLP [19].

Sarra R. R. et al. proposed a new classification model based on SVM for better prediction of heart disease using the Cleveland and Statlog datasets from the UCI Machine Learning repository. The χ^2^ statistical optimal feature selection method was used to improve the prediction accuracy of the model. The performance of the proposed model is evaluated against traditional classifier models using various performance metrics, and the results showed that the accuracy improved from 85.29% to 89.7% by applying the proposed model [20].

Malavika G. et al. investigated the use of ML algorithms to predict heart disease. The heart disease dataset from the UCI repository was used for this study. They used various ML algorithms, including LR, KNN, SVM, NB, DT, and RF, to predict heart disease, and their performances were compared. The results showed that RF (91.80%) had the highest accuracy in predicting heart disease, followed by NB (88.52%) and SVM (88.52%). The authors concluded that ML algorithms could be a useful tool in predicting heart disease and could potentially help doctors diagnose and treat patients more accurately [21].

Sahoo G. K. et al. compared the performance of LR, KNN, SVM, NB, DT, RF, and XG Boost Machine Learning models for predicting heart disease. The Cleveland heart disease dataset from the UCI ML repository was used to train the models. Comparing the results of the tested ML algorithms, the RF algorithm performed the best, with a classification accuracy of 90.16% [22].

The exploration of various ML techniques for predicting coronary artery disease is addressed in [23]. The study used a dataset of 462 medical instances, and nine features from the South African heart disease dataset. It consists of 302 healthy records and 160 records with coronary heart disease. In this study, the k-means algorithm, along with the synthetic minority oversampling technique, were used to solve the problem of imbalanced data. A comparative analysis of four different ML techniques, such as LR, SVM, KNN, and artificial neural network (ANN), can accurately predict coronary artery disease events from clinical data. The results showed that SVM had the highest accuracy performance (78.1%) [23].

In Ahmad G. N. et al.’s study, Cleveland, Hungarian, Switzerland, Statlog, and Long Beach VA datasets were combined to obtain a larger dataset compared to existing heart disease datasets. They compared the performances of LR, KNN, SVM, Nu-Support Vector Classifier (Nu-SVC), DT, RF, NB, ANN, AdaBoost, Gradient Boosting (GB), Linear Discriminants Analysis (LDA) and Quadratic Discriminant Analysis (QDA), algorithms for heart disease classification. In this study, the authors claimed that the best classification accuracy of 100% was achieved with the RF algorithm [24].

The main objective of this study is to use the metaheuristic method, such as the Jellyfish algorithm, to select the optimum features from the heart disease dataset and use it in the Machine Learning method to classify the healthy and non-healthy heart disease data. Some of the features do not have more efficiency in the classification of heart disease. The Jellyfish has some advantages, such as the high speed of convergency, and high accuracy to find the features. For this reason, this algorithm has been selected.

## 2. Material and Method

This paper presents a performance analysis of different ML techniques based on selecting the meaningful features of the dataset in the hope of improving heart disease prediction accuracy. In this study, the performance of different ML models such as ANN, DT, Adaboost, and SVM using the Jellyfish algorithm and feature selection for the prediction of heart disease was compared, aiming at obtaining the highest performance model. The Cleveland dataset used in this study was obtained from the Kaggle Machine Learning repository.

### 2.1. Dataset

The Cleveland heart disease dataset is commonly used for heart disease prediction with supervised Machine Learning. The Cleveland dataset is obtained from the Kaggle Machine Learning repository. The Cleveland dataset was collected for use in a study in the field of health research by the Cleveland Clinic Foundation in 1988. In the original of this dataset, 76 different features of 303 subjects were recorded. However, it is known that most researchers use only 14 of these features, including the target class feature. These features include age, gender, blood pressure, cholesterol, blood sugar, and many more health metrics. The original Cleveland dataset has five class labels. It has integer values ranging from zero (no presence) to four. The Cleveland dataset experiments have focused on just trying to discriminate between presence (Values 1, 2, 3, 4) and absence (Value 0). However, the number of samples for each class is not homogeneous (Values 0, 1, 2, 3, 4—samples 164, 55, 36, 35, 13). Researchers suggest that the five class features of this data set be reduced to two classes; 0 = no disease and 1 = disease. The target feature refers to the presence of heart disease in the subject. Table 1 shows the features included in the Cleveland heart disease dataset.

**Figure 1:**
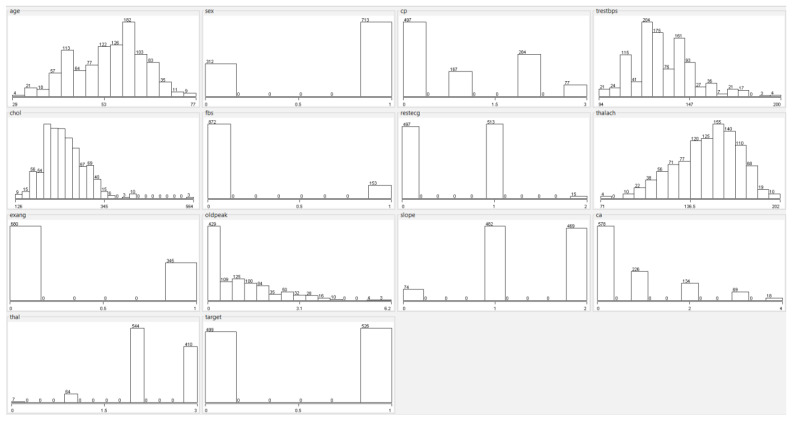
Histograms of features in the heart disease dataset.

In the original dataset, a total of 6 samples have null values; 4 samples in the “Ca (Number of Major Vessels)” feature and 2 samples in the “Thal (Thallium Heart Rate)” feature. Since null values are very few, these samples can be removed from the dataset. The dataset used in this study contains a total of 1025 samples. A total of 499 samples belong to the disease (1), and 526 of these samples belong to the no disease (0) class. Histograms of all features in the Cleveland heart disease dataset are shown in Figure 1.

### 2.2. Feature Selection and Dimension Reduce

The performance of ML models depends on the quality of the features used as input. As the number of features in the datasets increases, the prediction performance of the model decreases, and the computational costs increase. By reducing the number of features, the model can obtain more accurate results and work faster and more efficiently. ML models are designed according to the data used in the learning process. Selecting the best features makes the features learned by the model more generalizable. Thus, it makes the model work better with new data. Some features in the datasets are not important to the result and increase the computational complexity of the model. Removing unnecessary features reduces noise and helps the model achieve better results. Also, feature selection is important for understanding the nature of the dataset. Well-chosen features help people better understand the data. In this study, the Jellyfish algorithm was used to select the best features from the dataset.

**Figure 2:**
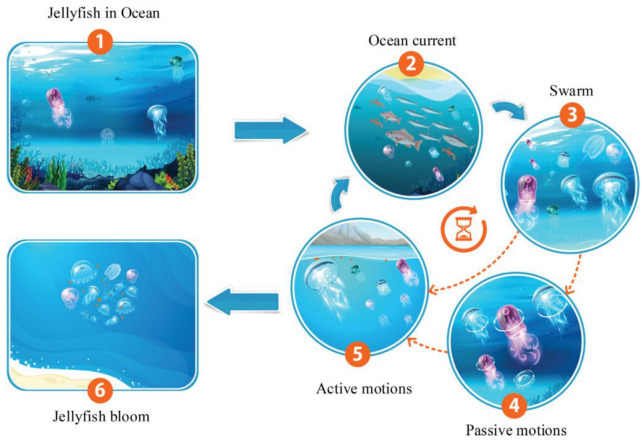
Jellyfish behaviors for modeling a jellyfish optimization algorithm [25].

Presented in 2021, the Jellyfish optimization algorithm is a type of swarm intelligence algorithm that is inspired by the food-finding behavior of jellyfish in the ocean. It is used to solve optimization problems, particularly in the field of engineering and computer science. According to the literature, the Jellyfish algorithm outperforms many well-known meta-heuristic algorithms in most real-world applications. In the Jellyfish algorithm, a group of artificial agents or particles, called “jellyfish,” move in a three-dimensional space, searching for the optimal solution to a problem. The algorithm is based on a set of rules that simulate the behavior of real-life jellyfish. The algorithm uses a combination of random and deterministic movements to explore the search space and exploit promising solutions. Each Jellyfish has a set of properties that are updated at each iteration, based on its own and the swarm’s best-known solutions. These properties include its position, velocity, and acceleration. The Jellyfish algorithm has been successfully applied to a range of optimization problems, including clustering, feature selection, and image segmentation. It has been shown to perform well in high-dimensional search spaces and can handle multiple objectives and constraints. Overall, the Jellyfish algorithm is a promising optimization technique that takes inspiration from nature to solve complex problems in a computationally efficient way. Figure 2 shows the behavior of jellyfish in the sea and the modeling of group movements [25].

The Jellyfish algorithm has the following three behaviors:

- A walker or jellyfish either follows the ocean current or moves within the group and can switch between the two modes intermittently;
- The jellyfish move in the ocean in search of food. They are more attracted to places where there is a lot of food;
- The amount of food found is determined by the location and function of the target.

Ocean waves in the sea contain nutrients that can attract jellyfish. The direction of current in the ocean can be defined with a vector and as in Equation (1):

(1) $$\vec{trend}=\frac{1}{nPop}.\sum {\vec{trend}}_{i}=\frac{1}{nPop}\sum \left(X^{*}-e_{c}X_{i}\right)$$

In this regard, $e_{c}$ is the absorption factor and a parameter. This equation can be extended as Equation (2):

(2) $$\vec{trend}=X^{*}-\frac{\sum e_{c}X_{i}}{nPop}=X^{*}-e_{c}\mu$$

In this equation, $X^{*}$ is the best jellyfish, and μ is the average population of the jellyfish. For simplicity, $df=e_{c}\mu$ can be assumed, and therefore this Equation can be more general and presented in Equation (3):

(3) $$\vec{trend}=X^{*}-\frac{\sum e_{c}X_{i}}{nPop}=X^{*}-df$$

The random distribution of jellyfish can be considered normal, as shown in Equations (4) and (5):

(4) $$df=\beta \times \sigma \times {rand}^{f}(0,1)$$

(5) $$\sigma ={rand}^{f}\left(0,1\right)\times \mu$$

**Figure 3:**
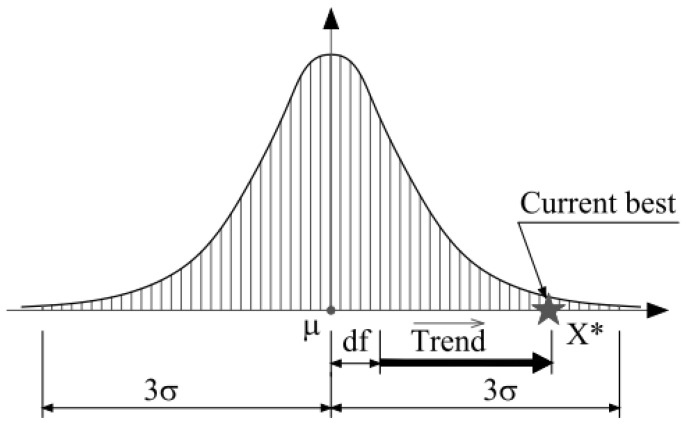
Normal distribution of jellyfish in the ocean [25].

In these relationships, σ is the standard deviation index of the distribution of jellyfish distribution. Figure 3 shows the normal distribution of jellyfish scattering around the mean point with the normal distribution.

**Figure 4:**
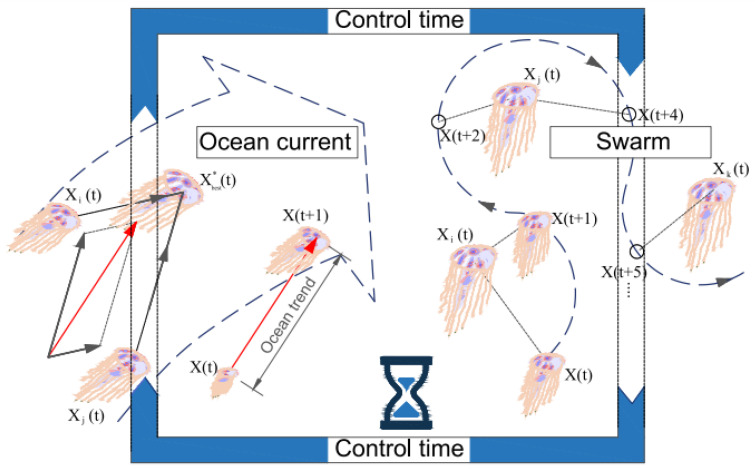
The movement of jellyfish in the ocean with the force of ocean movements and group movements [25].

Figure 4 depicts the displacement process of each jellyfish under the influence of ocean water force and under the influence of the jellyfish group.

The equations df and $e_{c}$ can be rewritten as Equations (6) and (7), respectively:

(6) df=β×rand(0,1)×μ

(7) $$e_{c}=\beta \times rand(0,1)$$

Now we can rewrite Equation (3) based on Equation (6) and present it in Equation (8):

(8) $$\vec{trend}=X^{*}-\beta \times rand(0,1)\times \mu$$

They are moved by water waves of jellyfish, the equation of which is given in Equation (9):

(9) $$X_{i}\left(t+1\right)=X_{i}\left(t\right)+rand(0,1)\times \vec{trend}$$

Equation (9) can be extended to Equation (10):

(10) $$X_{i}\left(t+1\right)=X_{i}\left(t\right)+rand(0,1)\times (X^{*}-\beta \times rand(0,1)\times \mu )$$

In this relation, β is a number greater than zero and is usually β=3. Jellyfishes also have group movements and usually have two passive and active movements. In the passive state, they search more around themselves. To model passive motion, Equation (11) is used to move them:

(11) $$X_{i}\left(t+1\right)=X_{i}\left(t\right)+\gamma .rand(0,1)\times (U_{b}-L_{b})$$

In this relation, γ is the coefficient of motion and is a positive number, and is usually set to 0.1. $U_{b}$ is the upper range of each dimension and $L_{b}$ is the lower range of one dimension. In the active behavior mode, a jellyfish-like $X_{i}$ randomly determines a jellyfish-like $X_{j}$, and there are two modes. If the merit of $X_{i}$ is greater than $X_{j}$, it uses Equation (12) to move; otherwise, Equation (13) is used:

(12) $$X_{i}\left(t+1\right)=X_{i}\left(t\right)+rand.(X_{j}\left(t\right)-X_{i}\left(t\right))$$

(13) $$X_{i}\left(t+1\right)=X_{i}\left(t\right)+rand.(X_{i}\left(t\right)-X_{j}\left(t\right))$$

Equation (14) is used to switch between ocean movements and group movements:

(14) $$c\left(t\right)=\left|\left(1-\frac{t}{Maxt}\right)\times (2.rand-1)\right|$$

**Figure 5:**
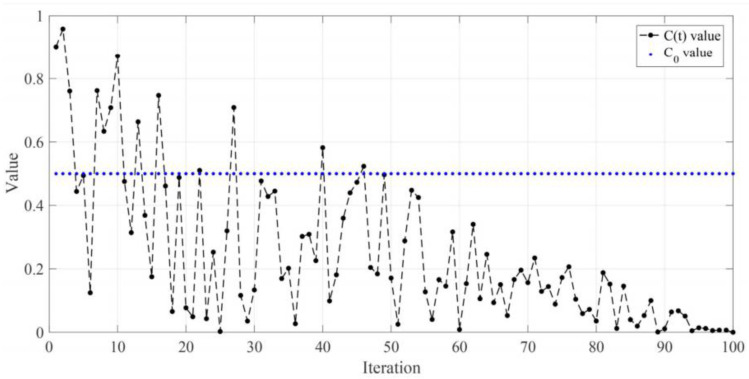
Random function to determine the type of motion of the type of force of ocean motions and group motions [25].

In this regard, t is the current iteration number of the algorithm, and Maxt is the maximum iteration counter. The diagram $c\left(t\right)$ Figure 5 is shown for an experiment. For each update, if the random number $c\left(t\right)$ is greater than 0.5, then the Jellyfish update is based on waves, and if it is less than 0.5, it is based on group movements.

### 2.3. Machine Learning Algorithms

Machine Learning refers to the use of computer algorithms that can learn to perform a particular task from sample data without explicitly programmed instructions. ML uses advanced statistical techniques to learn distinctive patterns from training data to make the most accurate predictions of new data. In applications such as disease prediction, ML models can often be developed using supervised learning methods. Supervised learning requires that training samples are correctly labeled. In its simplest form, the output is a binary variable with a value of 1 for patient subjects and 0 for healthy subjects. To obtain robust ML models, it is recommended to use balanced training samples from healthy and patient subjects. If several diseases are to be included in the ML model, the binary classification can be easily extended to the multi-class case. Therefore, supervised learning algorithms associate input variables with labeled outputs. In this study, we compare the performance of four different ML models using supervised learning, such as ANN, DT, Adaboost, and SVM.

ANN is one of the most basic and popular models of artificial neural networks. It is a network with two or more hidden layers and is often used to solve classification or regression problems. ANN consists of the input layer, one or more hidden layers, and output layers. Each layer contains one or more nodes (neurons). The input layer introduces data into the network and contains a node for each attribute. Hidden layers are layers used to process data. The output layer outputs the results and contains a node for each class in classification problems. ANN works by multiplying each node’s inputs by their weights, putting them into the activation function, and calculating the output. The activation function is the function that determines the output of each node, and non-linear functions such as sigmoid, ReLU, or tanh are often used. During the training process, the weights are randomly assigned, and then the weights are optimized using the backpropagation algorithm. The backpropagation algorithm minimizes the difference between the target outputs and the outputs of the network. ANN can be used for many different types of data and can be used in conjunction with other neural network models and extended to solve more complex problems.

The DT algorithm tries to classify data using a tree structure. The algorithm creates a set of decision rules that parse data according to a specific set of features. This set of decision rules is interconnected along the branches of the tree, forming a decision tree. Each branch corresponds to a decision rule, and each leaf node provides a class or value estimate. The algorithm helps to separate the classes by parsing the data. Each decomposition is accomplished by selecting a feature and dividing it among the values of that feature.

Adaboost (Adaptive Boosting) is an ML algorithm used to solve classification and regression problems. Adaboost algorithm works by combining weak classifiers (weak learners) into strong classifiers (strong learners). The algorithm starts by weighing each sample in the dataset. Initially, each sample has an equal weight. Then, a weak classifier is trained, and this classifier is selected considering the classification accuracy. The selected classifier reduces the weight of the samples it classifies as correct and increases the weight of the samples it classifies as incorrect. Next, a new weak classifier is trained with the weighted samples, and the process is repeated. This process continues until a predetermined number of weak classifiers are trained. Finally, a weighted vote is performed according to the classification accuracy of each weak classifier. As a result of this voting, a powerful classifier is obtained for classifying the given samples.

SVM is a preferred ML algorithm because it is resistant to outliers and gives good results when the data size grows. SVM represents data points in an n-dimensional space and tries to find the best hyperplane separating samples belonging to different classes. However, in some cases, data points cannot be separated linearly. In these cases, the SVM’s solution is found using more complex hyperplanes. The kernel trick allows the SVM to work with data that can be separated more easily in higher dimensional spaces by moving the data to higher dimensional spaces (kernel space). This allows it to perform the separation using more complex hyperplanes for the non-linearly separable dataset. The kernel trick works by using different kernel functions, especially the radial basis function (RBF) and the polynomial kernel. These kernel functions operate based on the properties of data points (distance, similarity, inner product, etc.) and allow the SVM to find an appropriate hyperplane that it can use to separate data in higher dimensional spaces.

### 2.4. Methodology

**Figure 6:**
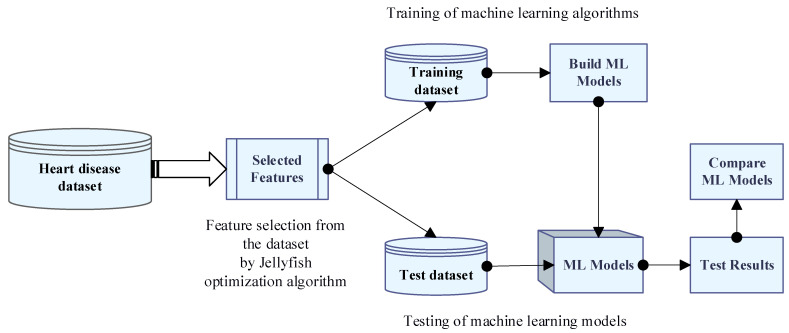
Flowchart of the proposed approach for heart disease prediction.

The main aim of this study is to provide clinicians with a tool to help them diagnose heart problems early. Therefore, it will be easier to effectively treat patients early and avoid serious consequences. In this study, the performance of different ML models using the Jellyfish algorithm and feature selection for heart disease prediction was compared, and we attempted to obtain the highest performance ML model. The summary of the proposed method is shown in Figure 6. As seen in Figure 6, firstly, the Jellyfish algorithm that was presented in 2021 was applied to the dataset to obtain the best features. The Jellyfish algorithm tries to find optimal solutions to various optimization problems by simulating the intelligent behavior of jellyfish. The Jellyfish algorithm does not get stuck in local minimums and reaches the global minimum faster than other optimization algorithms. The algorithm has attracted great attention around the world due to its simplicity of implementation, few parameters, and flexibility. Because of these advantages, the Jellyfish algorithm was preferred in this study to select the best features from the dataset. The Jellyfish algorithm has an effective feature selection role, and a binary version of it is used in this study. This algorithm starts with a population, which is a collection of potential solutions with the best features. The best features are selected for transfer to the next step in each iteration of the algorithm, which ultimately results in the best solution for the features. After creating a new dataset with the best features, this dataset was used for training four different classifiers such as ANN, DT, Adaboost, and SVM. The ML models obtained after the training were tested, and their performances were compared using metrics such as Accuracy, Sensitivity, Specificity, and Area Under Curve, and the ML model with the best performance was selected. A 10-fold cross-validation was used in the training and test phase of ML algorithms. This selected model has high performance in separating and classifying new data samples into two classes as no disease and diseased. In this study, MATLAB (version R2022a) was used for feature selection and classification.

## 3. Experimental Test Results

### 3.1. Performance Metrics

A table known as the confusion matrix is used to evaluate the performance of ML models. The confusion matrix is a table showing the difference between the actual and predicted classes. Each row of the confusion matrix represents an instance in the predicted class, while each column represents an instance in the real class (and vice versa). The confusion matrix usually contains four different terms: True Positive (TP), False Positive (FP), True Negative (TN), and False Negative (FN).

True Positive (TP) refers to situations where actual positives are correctly predicted as positives. False Positive (FP) refers to situations where actuals are incorrectly predicted as positives.

True Negative (TN) refers to situations where what is negative is correctly predicted as negative.

False Negative (FN) refers to situations in which true positives are incorrectly predicted as negatives.

Using these terms, performance metrics such as Accuracy, Sensitivity, Specificity, and Area Under Curve (AUC) are calculated. These evaluation criteria, commonly used in the context of binary classification tasks, are calculated as follows.

Accuracy: the proportion of true predictions (both true positives and true negatives) out of all predictions. It is calculated as (TP + TN)/(TP + TN + FP + FN).

Sensitivity (also called recall or true positive rate): the proportion of true positives out of all actual positive cases. It is calculated as TP/(TP + FN).

Specificity: the proportion of true negatives out of all actual negative cases. It is calculated as TN/(TN + FP).

Under the curve (AUC): It refers to the area under the ROC (Receiver Operating Characteristic) curve and takes a value between 0 and 1. If the value of AUC is 0, the classifier predicts all classes incorrectly, and if it is 1, the classifier correctly predicts all classes.

### 3.2. Test Results

In this section, the proposed method has been implemented on the test data, and the results have been compared with other ML methods such as ANN, Decision Tree, AdaBoost, and SVM. Also, the four different types of performance metrics, such as Sensitivity, Specificity, Accuracy, and Area Under Curve, have been calculated. In total, 70% of the data were selected for training and 30% for testing. Furthermore, other numbers of the training and testing data were selected and tested, but the best performance has been obtained from the mentioned percentages. The performance evaluation results of ML models without applying feature selection with the Jellyfish algorithm are given in Table 2.

**Figure 7:**
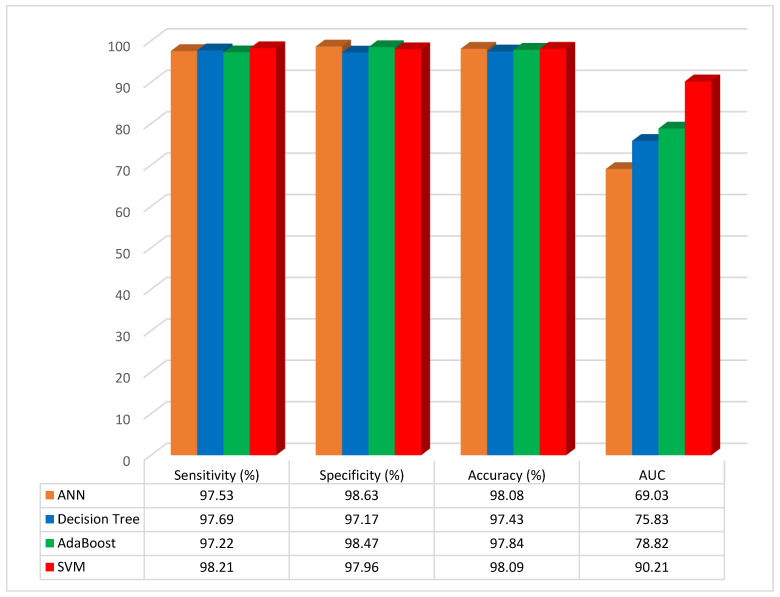
Graphical representation of performance evaluation results of ML models without feature selection.

According to the results of the studies, the classification accuracy of the ANN, DT, AdaBoost, and SVM classifier models was 98.08%, 97.43%, 97.84%, and 98.09%, respectively. The SVM classifier model was the most accurate when compared to the other ML models, and the accuracy rose to 98.09%. The results as graphical illustrations are shown in Figure 7.

The performance evaluation results of the ML models, when feature selection is applied with the Jellyfish optimization algorithm, are given in Table 3.

**Figure 8:**
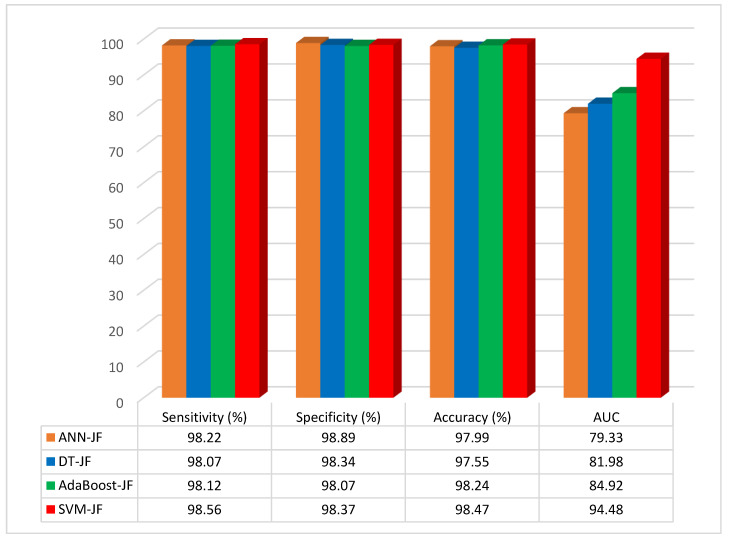
Graphical representation of performance evaluation results of ML models with feature selection.

According to the results of the studies, the accuracy of the ANN–JF, DT–JF, AdaBoost–JF, and SVM–JF was 97.99%, 97.55%, 98.24%, and 98.47%, respectively. The SVM-based Jellyfish approach was the most accurate when compared to the other methods, and the accuracy rose to 98.47% when feature selection was combined with the Jellyfish algorithm. The results as a graphical illustration are shown in Figure 8.

The method of combining feature selection based on the Jellyfish optimization algorithm and SVM has higher Area Under Curve values than the other methods. In this method, the best features can be selected by using the Jellyfish algorithm and the SVM method to classify the data more accurately than other ML methods.

Furthermore, a case comparison between the current study and references [26,27] has been conducted by the classification accuracy evaluation criteria, with the findings displayed in Table 4.

The suggested approach in this study achieves favorable outcomes in the evaluation criteria. The classification accuracy of its prediction of heart disease is also higher than that of some studies in the literature and comparable techniques.

As seen in Table 4, the proposed method reached 98.47% accuracy. This result shows that the optimum features can be used for heart disease diagnosis. The best features selected by Jellyfish improve the accuracy of results, because some of the features that are not selected by the Jellyfish algorithm can reduce the performance of the classification results. However, in classical methods such as Principal Component Analysis (PCA), some of the features that are not so important can be selected, which can reduce classifier model performance.

**Figure 9:**
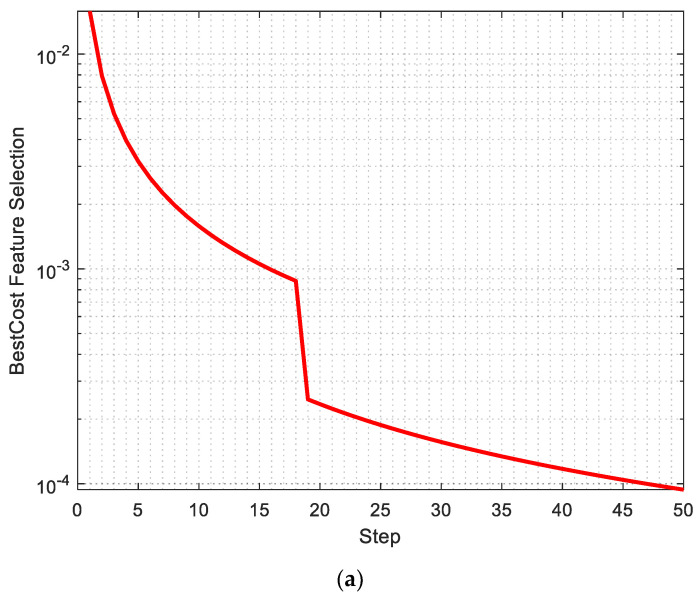

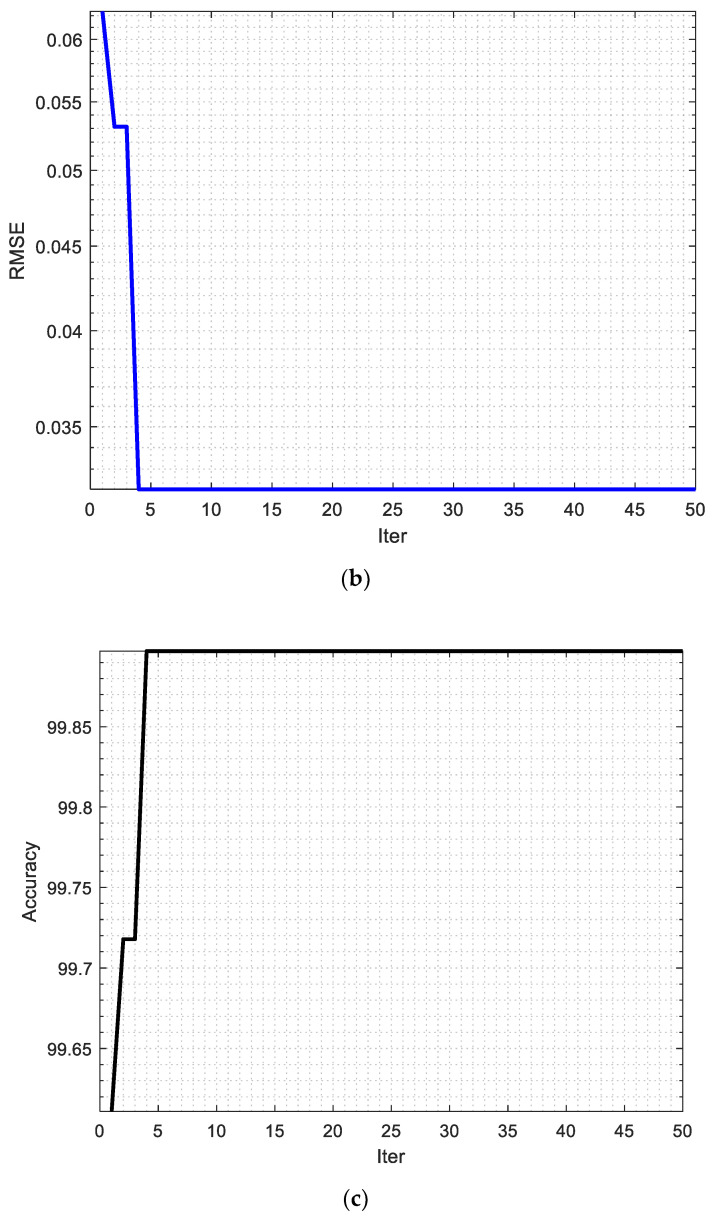
(**a**) Best cost of feature selection, (**b**) Root Mean Square Error, and (**c**) accuracy of the proposed method.

The best cost of feature selection, the Root Mean Square Error, and the accuracy of the proposed are shown in Figure 9a–c, respectively.

As seen in Figure 9a, the best cost of feature selection is obtained in 50 iterations, and this value is 0.0004, which is close to zero. Also, Figure 9b shows the Root Mean Square Error that reached 0.030 in the fourth iteration.

Heart Valve Disease refers to any condition that affects the heart valves. The heart has four valves, known as mitral, tricuspid, aortic, and pulmonary, which open and close to allow blood to flow in one direction through the heart. Heart Valve Disease occurs when one or more of the valves work improperly. When the valves are healthy, they keep blood flowing smoothly through the heart and body. But when the valves are diseased, they may not open and close properly, causing blood to back up or leak in the wrong direction. Procedures to repair or replace heart valves can include balloon valvuloplasty, surgical valve repair, or surgical valve replacement.

Heart Failure is a condition in which the heart is unable to pump enough blood to meet the body’s needs. The heart may be weakened, stiffened, or damaged, and is unable to efficiently circulate blood throughout the body. This can lead to fluid build-up in the lungs, legs, and other areas of the body. There are two main types of heart failure: systolic and diastolic. Systolic heart failure occurs when the heart’s ability to contract and pump blood is impaired, while diastolic heart failure occurs when the heart is stiff and unable to fill with blood properly. Heart failure can be caused by a variety of factors, including coronary artery disease, high blood pressure, heart valve disease, heart attack, and certain medications.

The findings show that, compared with previous approaches, the proposed strategy improves percent accuracy in heart disease diagnosis. The results of this study demonstrate the potential of artificial intelligence, particularly ML, to significantly influence heart disease diagnostic decisions. The steady increase in computing power and increased data availability through mobile apps and the digital transformation of the global healthcare system are driving the growth of artificial intelligence and ML further. Therefore, future research will continue to use these techniques to translate them into routine clinical practice, thus paving the way for improved diagnostic decision-making to suit the specific needs of individual patients.

Machine learning algorithms for the diagnosis of heart diseases may have significant potential in the medical diagnosis process. These algorithms can be trained on datasets to perform tasks such as diagnosing specific heart diseases, assessing risk factors, and recommending treatment options. However, the potential risks and problems of these applications should also be considered. Several aspects of this debate can be addressed:

Data quality and accuracy: The proposed algorithm requires sufficient and high-quality data to produce accurate and reliable results. Therefore, the datasets used should not contain incomplete, inaccurate, or misleading data. Especially in a field such as heart disease, misdiagnosis recommendations can be errors that can have serious consequences.

Understandability of the algorithm: It may be necessary to explain to doctors how the algorithm and its parameters work. If doctors do not understand the decision processes of the algorithm, they may find it difficult to fully trust its results.

Data privacy and security: Privacy and security concerns may arise when using patients’ medical data. It is important that the data is properly protected and protected from unauthorized access and malicious use. This should be considered during the implementation of algorithms into clinical practice.

Physician–patient relationship: Some patients may find it difficult to trust their doctors regarding a diagnosis or treatment recommendation made by the algorithm, or may be skeptical about the results of the algorithm. The proposed algorithm should only be considered as a tool to assist physicians in their decision-making process. It should not be perceived as interfering with doctors’ decision-making.

## 4. Conclusions

This study aimed to obtain a highly accurate and reliable intelligent medical diagnosis model based on ML with the Jellyfish optimization algorithm using the Cleveland data set for early prediction of heart disease. One of the important factors affecting the performance of an ML model is the number of features in the dataset used. Choosing the right features can help the model better understand the data and give more accurate results. Selecting the right features can improve the performance of the model, while selecting too many features can increase the complexity of the model and cause overfitting. Therefore, the number of features must be accurately determined. To avoid the overfitting problem due to the large number of features in the Cleveland dataset used in this study, the best features were selected from the dataset by using the Jellyfish algorithm. The Jellyfish algorithm is a swarm-based metaheuristic algorithm that can be used with ML methods to optimize hyperparameters. The optimum features obtained from the dataset were used in the training and testing stages of four different ML algorithms (ANN, DT, AdaBoost, and SVM). Then, the performances of the obtained models were compared. The results show that the accuracy rates of all ML models improved after the dataset was subjected to feature selection with the Jellyfish algorithm. The highest classification accuracy (98.47%) was obtained with the SVM model trained using the dataset optimized with the Jellyfish algorithm. The Sensitivity, Specificity, Accuracy, and AUC for SVM without using the Jellyfish algorithm were obtained at 98.21%, 97.96%, 98.09%, and 90.21%, respectively. However, by using the Jellyfish algorithm, these values have been obtained as 98.56%, 98.37%, 98.47%, and 94.48%, respectively.

## Figures and Tables

**Table 1:** List of features in the Cleveland heart disease dataset.

Order	Feature	Description	Feature Value Range
1	Age	Age in years	29 to 77
2	Sex	Gender	Value 1 = male Value 0 = female
3	Cp	Chest pain type	Value 0: typical angina Value 1: atypical angina Value 2: non-anginal pain Value 3: asymptomatic
4	Trestbps	Resting blood pressure (in mm Hg on admission to the hospital)	94 to 200
5	Chol	Serum cholesterol in mg/dL	126 to 564
6	Fbs	Fasting blood sugar > 120 mg/dL	Value 1 = true Value 0 = false
7	Restecg	Resting electrocardiographic results	Value 0: Normal Value 1: having ST-T wave abnormality (T wave inversions and/or ST elevation or depression of >0.05 mV) Value 2: showing probable or definite left ventricular hypertrophy by Estes’ criteria
8	Thalach	Maximum heart rate achieved	71 to 202
9	Exang	Exercise-induced angina	Value 1 = yes Value 0 = no
10	Oldpeak	Stress test depression induced by exercise relative to rest	0 to 6.2
11	Slope	The slope of the peak exercise ST segment	Value 0: upsloping Value 1: flat Value 2: downsloping
12	Ca	Number of major vessels	Number of major vessels (0–3) colored by fluoroscopy
13	Thal	Thallium heart rate	Value 0 = normal; Value 1 = fixed defect; Value 2 = reversible defect
14	Target	Diagnosis of heart disease	Value 0 = no disease Value 1 = disease

**Table 2:** Performance comparison of different ML models without the Jellyfish algorithm.

Model	Sensitivity (%)	Specificity (%)	Accuracy (%)	AUC (%)
ANN	97.53	98.63	98.08	69.03
Decision Tree	97.69	97.17	97.43	75.83
AdaBoost	97.22	98.47	97.84	78.82
SVM	98.21	97.96	98.09	90.21

**Table 3:** Performance comparison of different ML models when applying feature selection with the Jellyfish algorithm.

Model with JF	Sensitivity (%)	Specificity (%)	Accuracy (%)	AUC (%)
ANN with JF	98.22	98.89	97.99	79.33
DT with JF	98.07	98.34	97.55	81.98
AdaBoost with JF	98.12	98.07	98.24	84.92
**SVM with JF**	**98.56**	**98.37**	**98.47**	**94.48**

**Table 4:** Comparison of the approach proposed in this study with some studies in the literature in terms of classification accuracy.

Reference	Dataset	Accuracy (%)
[17]	Cleveland and Statlog heart dataset	89
[18]	Cleveland heart dataset	88.5
[19]	Cleveland heart dataset	94.6
[20]	Cleveland and Statlog heart dataset	85.29
[21]	Cleveland heart dataset	91.8
[22]	Cleveland heart dataset	90.16
[23]	South African heart dataset	78.1
**Proposed method**	**Cleveland heart disease dataset**	**98.47**

## Data Availability

No data were used to support this study.
